# Resurgence of Human Brucellosis in Khuzestan Province, Southwestern Iran: Epidemiological Trends From 2017 to 2024

**DOI:** 10.1155/ijm/8935628

**Published:** 2026-08-11

**Authors:** Mehran Varnasseri Ghandeali, Amirmansoor Varnasseri Ghandali, Homayoun Amiri, Nazafarin Varnasseri, Maryam Dadar

**Affiliations:** ^1^ Infectious and Tropical Diseases Research Center, Health Research Institute, Ahvaz Jundishapur University of Medical Sciences, Ahvaz, Iran, ajums.ac.ir; ^2^ Medical Department, Ahvaz Jundishapur University of Medical Sciences, Ahvaz, Iran, ajums.ac.ir; ^3^ Health Center, Ahvaz Jundishapur University of Medical Sciences, Ahvaz, Iran, ajums.ac.ir; ^4^ Independent Physician, Ahvaz, Iran; ^5^ Brucellosis Department, Razi Vaccine and Serum Research Institute (RVSRI), Agricultural Research, Education and Extension Organization (AREEO), Karaj, Iran, areo.ir; ^6^ Iran Veterinary Council, Tehran, Iran

**Keywords:** brucellosis, geographic heterogeneity, Iran, log-linear regression, One Health, temporal trends, zoonoses

## Abstract

Brucellosis is a neglected zoonosis with a considerable disease burden in livestock‐dependent areas. Khuzestan Province, southwestern Iran, exhibits diverse ecological and agricultural landscapes that may drive heterogeneous transmission. Knowledge of recent epidemiological trends is important for targeted control. We aimed to characterize the temporal and demographic patterns of human brucellosis in Khuzestan from 2017 to 2024, with emphasis on changes observed after 2021. We analyzed aggregated surveillance data for all laboratory‐confirmed human brucellosis cases reported in Khuzestan Province between 2017 and 2024. Crude annual incidence rates were calculated using population denominators linearly interpolated from the 2017 census and the 2023 official estimate. Log‐linear trend regression, performed using Joinpoint software, estimated the annual percentage changes (APCs) in incidence case counts. Poisson′s regression with population offset quantified period relative risks (RRs) comparing 2022–2024 with 2017–2021. Chi‐square tests assessed temporal shifts in distributions across age groups (15 categories), sex, residence (rural/urban/nomadic), and four geographical zones (Northern Highlands, Central Plains, Southern Coastal and Marshland, and Western Borderlands). A total of 3923 cases were reported. Crude incidence remained stable at 7.9–8.1 per 100,000 during 2017–2019, declined to 6.3–7.0 in 2020–2021, and then increased sharply to 12.3–14.7 in 2022–2024 (APC = +10.7*%*; 95% CI: 2.2–19.9; *p* = 0.019). Incidence during 2022–2024 was 1.66 times higher than during 2017–2021 (95% CI: 1.52–1.81; *p* < 0.001). The largest relative increases in reported case counts were in adults aged ≥ 70 years (APC = +21.1*%*; 95% CI: 11.1–32.2) and in adults aged 65–69 years (+20.0%, 9.9–31.1), whereas children aged 0–4 years showed no significant change. The male‐to‐female ratio shifted from 0.88 in 2017 to 1.22 in 2024 (*p* = 0.032), driven by a significant rise in male cases (APC = +11.5*%*, *p* = 0.011) against a nonsignificant female increase. Rural cases increased significantly (APC = +11.2*%*, *p* = 0.009), accounting for 47%–61% of annual cases. The Northern Highlands zone bore 62.7% of total cases (APC = +11.6*%*, *p* = 0.003); the Southern Coastal zone exhibited the steepest relative increase (APC = +15.8*%*, *p* = 0.020). Although statistically significant, changes in composition over time were small in magnitude (Cramér′s *V* = 0.06 − 0.12). Human brucellosis in Khuzestan Province has resurged dramatically since 2021, with a 66% increase in period risk. The epidemiological profile shifted to older adults, males, and people living in rural areas, with marked geographical heterogeneity. The results emphasize the need for targeted vaccination of livestock in high‐burden areas (Northern Highlands and Southern Coastal), enhanced surveillance in older age groups, and increased One Health approaches to reverse this trend.

## 1. Introduction

Brucellosis is the most common zoonotic disease worldwide, especially in low‐ and middle‐income countries, where livestock farming plays a key role in supporting rural livelihoods and ensuring food security [1]. It is caused by the *Brucella* species, and the disease is transmitted to humans through consumption of unpasteurized dairy products, direct contact with infected animals, or inhalation of contaminated aerosols. High‐incidence regions include the Middle East, North and East Africa, Central/West Asia, parts of Latin America, and Asia [2]. The World Health Organization calls brucellosis a “neglected zoonosis” that remains a major health problem. Approximately 2.1 million new human cases are estimated, based on a conservative, evidence‐based estimate, significantly higher than previously assumed [1]. Iran is among the highest burden countries for human brucellosis, with reported incidence rates that have fluctuated dramatically over recent decades. The Khuzestan Province has a wide range of geography, from the cool, livestock‐dense Zagros highlands in the north to the humid, industrialized coastal areas along the Persian Gulf in the south, and to the marshland‐bordered western boundary with Iraq [3]. These differences in ecology and population likely lead to different local transmission patterns, but few studies look at brucellosis trends across such diverse areas within a single endemic province. Within Iran, human brucellosis cases concentrate in cooler, livestock‐rich uplands and rural areas, whereas coastal and southern industrialized areas show lower risk [4]. In this study, we aim to conduct a comprehensive epidemiological analysis of all laboratory‐confirmed human brucellosis cases reported in Khuzestan Province from 2017 to 2024. Using eight consecutive years of surveillance data, we aimed to (i) delineate temporal trends in overall case counts and crude incidence rates using log‐linear regression analysis; (ii) compare reported case counts between the earlier (2017–2021) and later (2022–2024) study periods; (iii) assess whether the distribution of cases across age groups, sex, residence (rural/urban/nomadic), and four geographical zones changed significantly during the study period; and (iv) identify which population subgroups experienced the most rapid relative increases using age‐specific annual percentage changes (APCs). By integrating descriptive epidemiology, log‐linear regression analysis, Poisson′s period risk modeling, and chi‐square independence testing, we provide, to our knowledge, the first detailed provincial surveillance‐based temporal and geographic trend analysis of brucellosis in a high‐burden Iranian province spanning the critical period of 2021–2024.

## 2. Materials and Methods

### 2.1. Study Area and Data Source

This study was conducted in Khuzestan Province, southwestern Iran. Human brucellosis case data were obtained from the provincial health surveillance system for the years 2017–2024. All cases met the diagnostic criteria of the national health industry standard for brucellosis diagnosis, which require compatible clinical symptoms (e.g., fever, arthralgia, and sweating) and confirmation by serological tests (standard tube agglutination test, 2‐mercaptoethanol test, ELISA, and Coombs–Wright test) or by isolation of *Brucella* spp. Cases were aggregated annually by age group (0–4, 5–9, … and 70+ years), sex (male and female), area of residence (rural, urban, and nomadic), and geographical zone.

### 2.2. Population Denominators

The Iranian Statistical Center provided population data for Khuzestan Province. We had census counts for 1996 (3,746,772), 2006 (4,274,979), 2011 (4,531,720), and 2016 (4,710,509), as well as an official estimate for 2023 (5,074,000) [5]. To find the annual populations for 2017–2024, we used linear interpolation/extrapolation assuming constant annual population growth between the 2016 census and the official 2023 estimate. The annual increment over this period was (5,074,000 − 4,710,509)/7 = 51,927 persons per year. Using this increment, the estimated populations are as follows: 2017: 4,762,436; 2018: 4,814,363; 2019: 4,866,290; 2020: 4,918,217; 2021: 4,970,144; 2022: 5,022,071; 2023: 5,074,000; and by extrapolation, 2024: 5,125,927. The 2024 population was extrapolated assuming the same annual growth rate continued. Crude annual incidence rates (per 100,000 population) were calculated as (reported cases/population) × 100,000. A major limitation is that no age‐, sex‐, residence‐, or geographical zone–specific population denominators were available. Therefore, subgroup‐specific incidence rates could not be calculated. Consequently, all subgroup analyses were based on reported case counts and describe trends in reported disease burden rather than true population risk.

### 2.3. Geographical Categorization of Cities

Based on topography, river systems, and administrative proximity, we classified 22 cities and districts of Khuzestan into four geographical zones (Table 1). This categorization was developed to capture regional heterogeneities in brucellosis transmission.

**Table 1:** Geographical zones of Khuzestan Province used in the analysis.

Zone	Cities included	Geographic rationale
1. Northern Highlands (Zagros foothills)	Andimeshk, Andika, Izeh, Dehdez, Masjed Soleyman, Lali, Baghmalek, and Gotvand	These cities lie in the northern and eastern mountains along the Zagros range. They share cooler climates and higher elevations and are upstream on the Karun and Dez rivers. Traditional livestock farming is common.
2. Central Plains (Ahvaz core & surroundings)	Ahvaz, Bavi, Karun, Hamidiyeh, Ramhormoz, Haftgel, Shush, and Karkheh	This zone forms the central agricultural plain around the provincial capital. Ahvaz is the main urban center. Bavi, Karun, and Hamidiyeh are former parts of Ahvaz County.
3. Southern Coastal & Marshland (Persian Gulf influence)	Bandar‐e Mahshahr, Omidiyeh, and Hendijan	These cities are located near the Persian Gulf coast or along tidal creeks. They have a humid, warm climate and significant petrochemical/oil industries. Livestock patterns differ from the highlands.
4. Western Borderlands (marsh & desert fringe)	Dasht‐e‐Azadegan, Ramshir, and Hoveyzeh	Dasht‐e‐Azadegan lies west of Ahvaz, bordering Iraq, and is bordered by vast marshlands (Hoor al Azim). Ramshir is a smaller city southwest of Ramhormoz, often grouped with the western border due to similar arid conditions and tribal populations.

*Note:* Case counts for each zone were aggregated annually from the original city‐level data.

### 2.4. Statistical Analyses

#### 2.4.1. Descriptive Analysis

Annual case counts and proportions were calculated for the whole province, as well as stratified by age group (15 categories), sex (male/female), area of residence (rural/urban/nomadic), and geographical zone (Northern Highlands, Central Plains, Southern Coastal and Marshland, and Western Borderlands). For each year from 2017 to 2024, crude incidence rates per 100,000 population were computed by dividing the annual number of reported cases by the corresponding interpolated total population estimate (derived from linear interpolation between the 2016 census and the 2023 projection, as detailed in Section 2.2). We used descriptive statistics to look at how case counts and incidence rates changed over time. We also used proportions and bar charts to compare how cases were spread out across categories over the years.

#### 2.4.2. Log‐Linear Regression Analysis

Log‐linear regression analysis was performed using Joinpoint software Version 4.8.0.1 (National Cancer Institute, United States). The model is specified as ln(*y*) = *α* + *β* *x* + *ε*, where *y* is the annual number of reported cases (or crude incidence rate per 100,000), *x* is the calendar year, *α* is the intercept, *β* is the slope coefficient, and *ε* is the random error. Due to the short time series (8 years), log‐linear regression was implemented in the Joinpoint software for all outcomes to avoid overfitting and unstable breakpoint estimation. Results from these models should be interpreted with caution, given the limited number of data points. The APC was calculated as 100 × (*e* ^*β*^ − 1), and its 95% confidence interval (CI) was derived from the model; an APC was considered statistically significant if the 95% CI did not include zero.

We applied log‐linear trend regression to the following outcomes: total annual case counts and the crude incidence rate (overall), age‐specific case counts (15 age groups), case counts by sex (male and female), case counts by residence (rural, urban, and nomadic), and case counts by each of the four geographical zones (Northern Highlands, Central Plains, Southern Coastal and Marshland, and Western Borderlands). Because subgroup population denominators by age, sex, residence, and geographical zone were unavailable, subgroup‐specific incidence rates could not be calculated. Therefore, log‐linear regression analysis for these variables was performed using reported case counts. These analyses describe temporal changes in reported disease burden and should not be interpreted as changes in population risk.

#### 2.4.3. Period Relative Risk (RR)

To evaluate temporal changes in disease occurrence, we compared the earlier period (2017–2021) with the later period (2022–2024), based on the observed increase in incidence after 2021. Using a Poisson regression model with the log of population as an offset, we estimated RR and 95% CIs in R Version 4.2.0. The model was set up as log (cases) = log(population) + *α* + *β* · *I*(period), where *I*(period) is an indicator variable that equals 0 for the reference period (2017–2021) and 1 for the later period (2022–2024). We then calculated the RR for the later period as exp(*β*).

#### 2.4.4. Association Between Categorical Variables and Year

To assess whether the distribution of brucellosis cases across demographic and geographic categories changed over the study period, we employed Pearson′s chi‐square test of independence for each contingency table: age group (15 levels) × year (8 levels), sex (2 levels) × year (8 levels), residence (3 levels) × year (8 levels), and geographical zone (4 levels) × year (8 levels). The strength of any significant association was quantified using Cramér′s *V*, interpreted as follows: 0.0–0.1, negligible; 0.1–0.3, weak; 0.3–0.5, moderate; and > 0.5, strong. For each of the 15 age groups (0–4, 5–9, …, and 70+ years), we plotted annual case counts for 2017–2024 and calculated the APC using log‐linear regression analysis. Given the absence of age‐specific population denominators, age‐standardized incidence rates could not be computed; therefore, all age‐specific analyses are based on reported case counts and proportions rather than incidence rates. Specifically, we conducted (i) calculation of year‐by‐year proportions of male versus female cases and of rural, urban, and nomadic cases, (ii) Pearson′s chi‐square tests of independence to assess whether the distribution of cases by sex and by residence changed significantly across years, and (iii) log‐linear regression analysis on case counts for each sex and each residence category separately to estimate APCs and their 95% CIs.

#### 2.4.5. Visualization and Software

All figures were generated in Python 3.9 using Matplotlib (Version 3.5.0) and Seaborn (Version 0.11.2). The main figures included a heat map displaying age‐specific case counts by year, line plots showing observed case counts and fitted log‐linear trends with 95% CIs for each of the four geographical zones, a stacked area chart illustrating the percentage distribution of cases by residence (rural, urban, and nomadic) with a secondary *y*‐axis for the total case trend, and a bar plot of period RRs with 95% CIs. Log‐linear regression analysis was performed using Joinpoint 4.8.0.1 (NCI, United States). All other statistical analyses (chi‐square, Poisson′s regression, and linear interpolation for population estimation) were conducted using R Version 4.2.0 (R Foundation for Statistical Computing, Vienna, Austria) with packages ggplot2, epitools, and reshape2.

## 3. Results

### 3.1. Overall Trends and Crude Incidence

**Figure 1:**
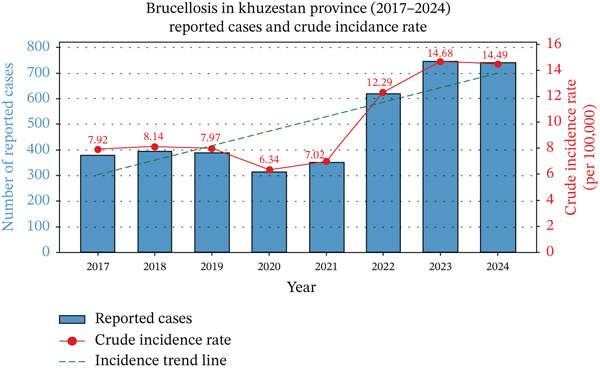
Annual reported brucellosis cases and crude incidence rate in Khuzestan Province, 2017–2024. Blue bars represent the annual number of reported brucellosis cases (left *y*‐axis), with values displayed above each bar. The red line with circular markers shows the crude incidence rate per 100,000 population (right *y*‐axis), with corresponding values displayed above each point. After 2021, the graph shows that both the number of cases and the incidence rate increased rapidly. There were 745 cases in 2023, for a rate of 14.68 per 100,000 people.

From 2017 to 2024, 3923 cases of brucellosis confirmed by clinical and serological tests were reported in Khuzestan Province. The annual number of cases was relatively stable from 2017 to 2019, with 377, 392, and 388 cases, respectively. Cases then declined to a low of 312 in 2020 and 349 in 2021, before increasing sharply to 617 in 2022, 745 in 2023, and 743 in 2024. The crude incidence rate per 100,000 population followed an identical pattern: 7.9–8.1 in 2017–2019, 6.3 in 2020, 7.0 in 2021, and then a dramatic rise to 12.3 (2022), 14.7 (2023), and 14.5 (2024) (Figure 1). Log‐linear trend regression yielded a statistically significant APC of +10.7% (95% CI: 2.2%–19.9%; *p* = 0.019), confirming a consistent increase in reported incidence over the 8‐year period.

### 3.2. Age‐Specific Trends

Across all 15 age groups, case counts increased over the study period, although the magnitude of increase varied substantially (Table 2). Older adults showed the greatest increase in reported case counts. The highest absolute numbers were consistently observed among adults aged 35–54 years. In contrast, the steepest relative increases, measured by the APC, occurred in the oldest age groups: 70+ years (APC = +21.1*%*; 95% CI: 11.1%–32.2%; *p* = 0.001), 65–69 years (APC = +20.0*%*; 95% CI: 9.9%–31.1%; *p* = 0.002), and 40–44 years (APC = +16.6*%*; 95% CI: 11.0%–22.5%; *p* < 0.001). In contrast, children aged 0–4 years exhibited a stable trend (APC = +4.0*%*; 95% CI: −16.9% to 30.2%; *p* = 0.640).

**Table 2:** Annual percentage change (APC) in reported cases by age group, Khuzestan 2017–2024.

Age group	APC (%)	95% CI	*p* value
0–4	4.0	(−16.9, 30.2)	0.640
5–9	12.0	(2.0, 23.2)	0.028
10–14	8.1	(1.9, 14.7)	0.018
15–19	15.2	(8.4, 22.4)	0.001
20–24	7.6	(−2.2, 18.5)	0.115
25–29	5.3	(−5.5, 17.2)	0.298
30–34	8.8	(2.3, 15.8)	0.016
35–39	13.5	(9.1, 18.1)	< 0.001
40–44	16.6	(11.0, 22.5)	< 0.001
45–49	12.2	(3.3, 21.9)	0.014
50–54	13.6	(5.8, 22.1)	0.004
55–59	11.4	(2.1, 21.5)	0.023
60–64	15.1	(2.9, 28.9)	0.022
65–69	20.0	(9.9, 31.1)	0.002
70+	21.1	(11.1, 32.2)	0.001

**Figure 2:**
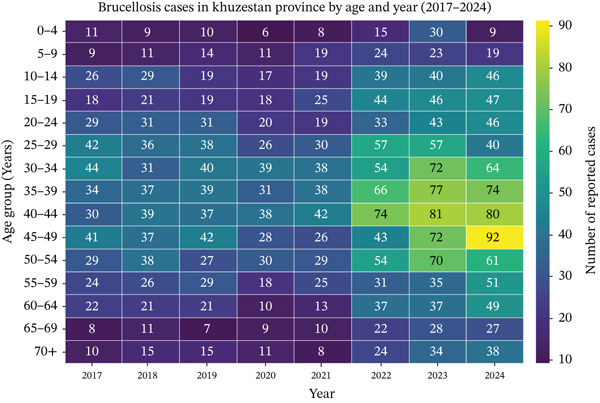
Age‐specific distribution of reported brucellosis cases in Khuzestan Province, 2017–2024. The heat map displays brucellosis cases by age (0–4 to 70+) and year (2017–2024). Cells range from light yellow (fewer cases) to dark blue (more cases), with exact numbers shown. Middle‐aged adults (35–54) report most cases, with a rise after 2021 across most ages.

The age‐specific heat map (Figure 2) visually confirms the post‐2021 increase across nearly all age groups, with a prominent dark band at ages 35–54. Because subgroup population denominators were unavailable, these findings reflect changes in reported case counts rather than age‐specific incidence rates.

### 3.3. Sex Distribution

**Figure 3:**
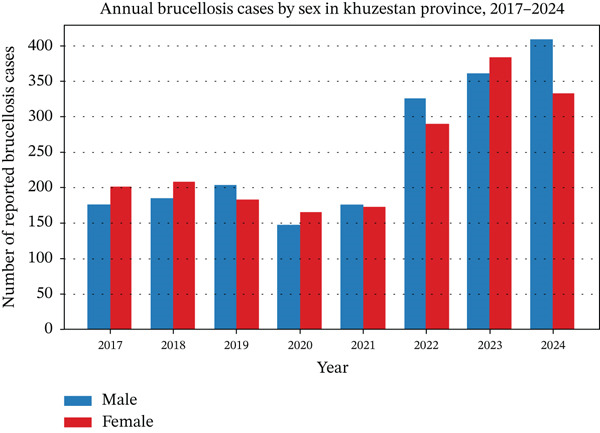
Annual brucellosis cases by sex in Khuzestan Province, 2017–2024. The figure shows the number of reported brucellosis cases among males (blue) and females (red) for each year from 2017 to 2024.

There were 3923 reported cases, with 49.4% (1938) in females and 50.6% (1985) in males. The male‐to‐female ratio varied substantially by year: 0.88 in both 2017 and 2018, then increased to 1.12 in 2019, fell to 0.89 in 2020, reached approximately parity at 1.02 in 2021, rose to 1.13 in 2022, decreased slightly to 0.94 in 2023, and peaked at 1.22 in 2024. From 0.88 in 2017, the ratio increased to 1.22 in 2024, indicating the relative increase in male cases during the study period (Figure 3). A chi‐square test of independence showed a statistically significant association between sex and year (*χ* ^2^ = 15.36, df = 7, *p* = 0.032), indicating that the distribution of sex changed over the study period. Log‐linear regression analysis showed a significant increase in male cases with an APC of +11.5% (95% CI: 3.5%–20.2%; *p* = 0.011). Female cases also increased, but the trend was not statistically significant (APC = +8.4*%*; 95% CI: −0.7% to 18.6%; *p* = 0.067), suggesting a more consistent rise among males. Because subgroup population denominators were unavailable, these findings reflect changes in reported case counts rather than sex‐specific incidence rates.

### 3.4. Residence Distribution

**Figure 4:**
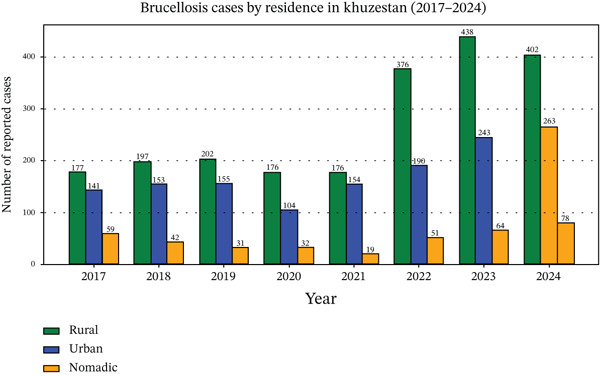
Annual brucellosis cases by residence in Khuzestan Province, 2017–2024. Rural (green line/bars), urban (blue), and nomadic (orange). Rural areas always had the most cases, and the number of cases rose sharply after 2021. Urban cases also rose but to a lesser extent, whereas nomadic cases remained low throughout the study period. The *y*‐axis represents the annual case count, and the *x*‐axis indicates the calendar year. Data points are connected by lines to illustrate temporal trends.

Rural areas accounted for the most cases each year, with yearly shares ranging from 47% to 61%. Urban areas contributed the second largest share (31%–44%), and nomadic areas contributed the least (5%–16%) (Figure 4). The share of rural cases increased from 47.0% in 2017 to 60.9% in 2022 and decreased to 54.1% in 2024. The share of nomadic cases decreased from 15.6% (2017) to 5.4% (2021) and recovered somewhat to 10.5% in 2024. A chi‐square test of independence (residence × year) gave *χ* ^2^ = 56.12 (*d* *f* = 14, *p* < 0.001), indicating a statistically significant but relatively weak shift in the distribution across years. Joinpoint regression produced APCs of +11.2% for rural (95% CI: 3.7%–19.2%; *p* = 0.009), +6.6% for urban (95% CI: −4.8% to 19.3%; *p* = 0.224), and +4.7% for nomadic (95% CI: −9.2% to 20.9%; *p* = 0.467). Only the rural increase was statistically significant. Because subgroup population denominators were unavailable, these findings reflect changes in reported case counts rather than residence‐specific incidence rates.

### 3.5. Geographical Zone Analysis

**Figure 5:**
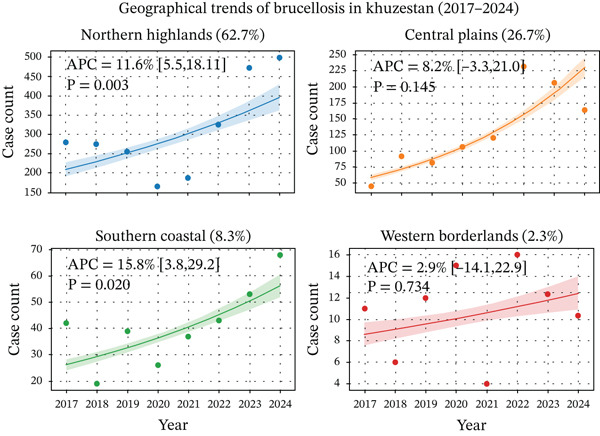
Geographical variation in brucellosis case trends across Khuzestan Province, 2017–2024. Each panel shows the annual reported case counts (circles), fitted log‐linear trend (solid line), and 95% confidence band (shaded area) for one of four zones. The percentage in parentheses indicates each zone′s share of total provincial cases (62.7%, 26.7%, 8.3%, and 2.3%). The APC, with 95% CI and *p* value, is displayed in each panel. Significant increases (*p* < 0.05) were observed in the Northern Highlands and Southern Coastal zones.

Marked regional differences emerged (Table 3). The Northern Highlands (Zagros foothills) recorded the highest case counts each year, accounting for 62.7% of the provincial total over the 8‐year span. Its numbers increased from 279 in 2017 to 499 in 2024, with an APC of 11.6% (95% CI: 5.5%–18.1%; *p* = 0.003). The Southern Coastal and Marshland zone, despite contributing only 8.3% of all cases, recorded the largest relative increase (APC = +15.8*%*; 95% CI: 3.8%–29.2%; *p* = 0.020). The Central Plains showed a positive but nonsignificant trend (APC = +8.2*%*; 95% CI: −3.3% to 21.0%; *p* = 0.145). The Western Borderlands remained low and flat (APC = +2.9*%*; 95% CI: −14.1% to 22.9%; *p* = 0.734). A chi‐square test for independence (zone × year) gave *χ* ^2^ = 143.6 (df = 21, *p* < 0.001), though the association was weak (Cramér′s *V* = 0.110). All reported cases were assigned to one of the four analytical zones (Figure 5). Because subgroup population denominators were unavailable, these findings reflect changes in reported case counts rather than geographical incidence rates.

**Table 3:** Annual brucellosis case counts by geographical zone, Khuzestan 2017–2024.

Zone	2017	2018	2019	2020	2021	2022	2023	2024	Total (%)	APC (%)
Northern Highlands	279	275	255	165	187	326	472	499	2458 (62.7)	**11.6**
Central Plains	45	92	82	106	121	232	206	164	1048 (26.7)	8.2
Southern Coastal	42	19	39	26	37	43	53	68	327 (8.3)	**15.8**
Western Borderlands	11	6	12	15	4	16	14	12	90 (2.3)	2.9
Total	377	392	388	312	349	617	745	743	3923	—

*Note:* APC in bold indicates a statistically significant increase (*p* < 0.05).

### 3.6. Period RR

**Figure 6:**
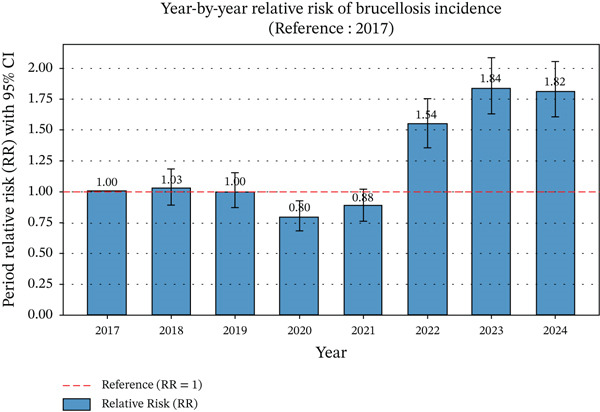
Year‐by‐year period relative risk (RR) of brucellosis incidence in Khuzestan Province, 2017–2024 (reference year: 2017). Bars represent the relative risk (RR) of brucellosis incidence for each year compared with the baseline year 2017 (RR = 1, red dashed line). Error bars indicate 95% confidence intervals calculated using the Poisson approximation. An RR above 1 indicates a higher incidence than in 2017, whereas an RR below 1 indicates a lower incidence.

Based on the observed increase in incidence after 2021, we compared the earlier period (2017–2021) with the later period (2022–2024). Poisson′s regression with a population offset estimated that the incidence during 2022–2024 was 1.66 times higher than during 2017–2021 (RR = 1.66; 95% CI: 1.52–1.81; *p* < 0.001). This comparison reflects a predefined period contrast rather than a formally identified change point. The temporal trend analysis using Poisson′s regression with calendar year as a continuous variable showed a significant increase in crude incidence during the study period (*p* < 0.001). Figure 6 shows the RRs per year with 2017 as the reference year. The highest RR was in 2023 (RR = 1.85; 95% CI: 1.64–2.08) and the lowest in 2020 (RR = 0.80; 95% CI: 0.69–0.93) (Figure 6).

### 3.7. Association Between Categorical Variables and Year

The chi‐square tests of independence (Table 4) indicated that the distribution of brucellosis cases across age groups, sex, residence, and geographical zone each changed significantly over the study period (all *p* < 0.05). However, the strength of these associations (Cramér′s *V*) was weak for all variables (range 0.09–0.12), suggesting that while demographic and geographic profiles shifted, the overall composition changed only modestly from year to year.

**Table 4:** Results of chi‐square tests for independence between categorical variables and year.

Variable	*χ* ^2^	*d* *f*	*p*value	Cramér′s *V*
Age group	204.6	98	< 0.001	0.107
Sex	15.36	7	0.032	0.063
Residence	56.12	14	< 0.001	0.119
Geographical zone	143.6	21	< 0.001	0.110

*Note:* All *p* values are two‐sided. Cramér′s *V* measures the strength of association (0.0–0.1, negligible; 0.1–0.3, weak).

## 4. Discussion

In this study, we observed a significant increase in the number of reported cases since 2021, with the crude incidence rate almost doubling from 7.0 per 100,000 in 2021 to 14.7 per 100,000 in 2023, with an average annual increase of 10.7%. This resurgence has been associated with distinct age, sex, residence, and geographical patterns, providing important insights into the underlying transmission dynamics and potential drivers of the outbreak. The crude incidence rates reported in Khuzestan before 2021 (approximately 6.3–8.1 per 100,000) are generally similar to the national average in Iran, estimated in some meta‐analyses to be 19.91–24.43 per 100,000 population but with considerable variation by province, being highest in western provinces such as Lorestan and Hamedan [6]. Another study also reported that the average annual incidence of the disease in Iran was 19.91 per 100,000. The three provinces with the highest cumulative prevalence were Lorestan, Hamedan, and Kurdistan, whereas the three with the lowest were Hormozgan, Tehran, and Bushehr [7]. However, this study presented a middle brucellosis prevalence in Khuzestan, in the 14.64–75 per 100,000 range with coldspots in the south/southwest, which is consistent with our findings. Likewise, Khuzestan was categorized as “low” incidence (0–10 per 100,000), with Bushehr/Hormozgan, opposite to western highs such as Lorestan [8].

At the national level, an ecological study using Joinpoint regression reported that the APC for human brucellosis incidence increased more strongly between 2009 and 2014 (17.1%) and then decreased from 2014 to 2018 (−9.2%) [7], a much smaller increase than the +10.7% observed in Khuzestan. This indicates that the rise in Khuzestan after 2021 is not just a reflection of the national trend but rather a separate local phenomenon. Several studies have demonstrated a decrease in brucellosis incidence in Iran after 2015. A national study on brucellosis from 2009 to 2017 reported the highest incidence in 2014 (25.89 per 100,000), followed by a decline [9]. Similarly, a study in Andika City (southwest Iran) covering 2014–2021 concluded that the incidence appeared to be decreasing [10]. Our study, however, shows a sharp reversal of this declining trend in Khuzestan from 2022 onwards, indicating that local factors may have outweighed the national downward trend. Globally, brucellosis is endemic in many low‐ and middle‐income countries, with Iran, Syria, Kyrgyzstan, Mongolia, Algeria, and Kenya reporting some of the highest incidence rates [2, 11]. The post‐2021 resurgence observed in Khuzestan may be driven by several plausible factors, although definitive causal inference is not possible with aggregated surveillance data. These are potential gaps in livestock vaccination coverage (especially of adult small ruminants), increased informal livestock trading, and movement of animals from high‐burden highlands to other zones following the COVID‐19 pandemic. Recent national reports have described persistent brucellosis in vaccinated herds and poor coverage of the traditional farming systems that prevail in the Northern Highlands [12, 13].

The present study also revealed a striking disproportionate increase in cases among older adults, especially those aged ≥ 65 years. Although the highest absolute number of cases was consistently seen among adults aged 35–54 years, the steepest relative increases (in terms of APC) were seen in the 70+‐year group, followed by 65–69 years. In contrast, children aged 0–4 years showed a stable trend. This age profile differs from that of previous studies in Iran, which have typically reported a higher incidence among younger adults [9, 14]. In a retrospective study in southwest Iran (2014–2021), the highest incidence was in the 21–30‐year age group (21%) [10]. Similarly, a national analysis from 2009 to 2017 estimated that 36.2% of cases occurred in the 25–44‐year age group and 16.7% in the 16–24‐year group [9]. In Behbahan County, the highest prevalence was observed in the 20–29‐year age group [15]. A study in Andimeshk covering 2001–2016 reported a mean age of 31.19 years, again indicating a predominance of younger adults [16].

Reported case counts increased most substantially in older age groups, but age‐specific incidence rates could not be estimated because of lack of age‐specific population denominators. These findings should, therefore, be interpreted with caution and not be taken as evidence of a disproportionate increase in disease risk in older adults. We cannot rule out other explanations, including changes in the population age structure, healthcare‐seeking behavior, and surveillance or reporting practices.

The shift toward older age groups in the present study could reflect changes in exposure patterns: Older adults in rural areas may have greater contact with livestock as younger family members migrate to urban centers for employment and increase their consumption of raw dairy products. Furthermore, the phenomenon may be related to decreased immunity in older populations that may have been exposed to different *Brucella* strains earlier in life.

The overall sex distribution in our study was nearly balanced, with 49.4% female and 50.6% male cases over the 8‐year period. However, the male‐to‐female ratio changed significantly over time, increasing from 0.88 in 2017 to 1.23 in 2024. Log‐linear trend analysis showed a pattern of a male‐predominant increase after 2021 that was an important departure from earlier literature. Previous studies in Iran have reported mixed findings regarding sex distribution. A study in Mazandaran Province reported an age‐standardized incidence rate of 15.6 per 100,000 in men, compared with 10.4 in women [17]. In Khorasan Razavi Province, over 84% of cases in certain clusters were male. However, other studies have reported a female predominance [18]. In Andika, 58% of patients were female [10]. Another study in Ilam Province (western Iran) found that 56.3% of cases were male and 43.7 were female [19]. A study in Andimeshk (within Khuzestan Province) reported a nearly equal sex distribution, with a male‐to‐female ratio of 1.02:1 [16]. The shift toward male predominance in our data may reflect changes in occupational exposure patterns.

Rural areas consistently accounted for the majority of cases throughout the study, and the rural case count showed a significant increase. The rural predominance is consistent with the extensive literature on brucellosis as a disease of agricultural and pastoral communities. A national analysis from 2009 to 2017 reported that 75.7% of cases were rural residents [9]. In western Iran, 95.2% of human cases were living in rural areas [20]. In Khorasan Razavi Province, the majority of patients in high‐risk clusters were rural residents [18]. The lower proportion of rural areas in our study compared with national figures may be due to the special characteristics of Khuzestan, which has large urban centers and a nomadic population. However, the steep upward trend in rural areas since 2021 (APC: +11.2%) suggests that the resurgence is being driven by transmission in rural areas, probably as a result of increased movements of livestock, breakdowns in vaccination programs, or expanded informal livestock trading. Nomadic cases remained stable and contributed the least proportion each year, although they were identified as high risk because of close contact with livestock, limited access to healthcare, and consumption of unpasteurized dairy products [21]. The lack of a substantial increase in nomadic cases in Khuzestan could be due to underreporting, changes in nomadic migration patterns during the study period, or real differences in transmission dynamics between settled rural populations and migratory pastoralists.

The geographical categorization developed in this study revealed marked regional heterogeneity in brucellosis transmission. Most cases were reported from the Northern Highlands (Zagros foothills) with a significant increasing trend. The Southern Coastal and Marshland zone had the highest relative increase, although it contributed only 8.3% of the cases. Meanwhile, the western borderlands were relatively stable. These results are consistent with the known spatial epidemiology of brucellosis in Iran, with the highest risk areas being in the western provinces and lower risk areas being generally in the central and southern regions [7]. Hotspots were observed in the west and northwest, whereas coldspots were observed in the south and southwest. The Northern Highlands of Khuzestan (part of the Zagros range) aligns with this western high‐risk corridor, providing a plausible explanation for the high case burden observed in this zone [7]. The Southern Coastal zone was previously considered at lower risk but had the highest relative increase. This increase may reflect changes in livestock management, livestock movement, or improved case ascertainment post‐2021. However, the trend in the Central Plains was not significant, and the trend in the Western Borderlands was flat, possibly due to lower population, underreporting, or true lower transmission [4]. There are hypotheses on the effect of the pandemic on reporting and transmission of human brucellosis. A study in Albania noted a decrease of 4.2 in 2020 due to animal vaccination, and similar trends were observed in other countries during healthcare disruption [22]. The initial decrease in cases could represent reduced access to healthcare or true decreases, but the sharp increase indicates resurgence. A study in China predicted increasing trends post‐pandemic of COVID‐19 [23]. Economic pressures in Iran may have increased informal livestock trading or reduced access to vaccines. The Human Development Index (HDI) has been shown to have an inverse association with brucellosis incidence at the national level in Iran, suggesting that socioeconomic development is protective [24]. Changes in livestock vaccination, particularly sheep and goats, may also have been a factor, as animal disease control reduces the number of human cases [25]. However, climate factors may have affected transmission, particularly in the Northern Highlands where the colder climate and traditional farming methods are prevalent [4].

The greatest RR was observed in 2023 compared with 2017 and the lowest in 2020. The overall increases in all age groups indicate that control efforts of the disease should be targeted at both the animal reservoirs and human exposure pathways. Priority interventions should include intensified livestock vaccination in the Northern Highlands and Southern Coastal zones, intensified surveillance and public health education, and better coordination between the human and veterinary sectors.

Several factors from the livestock point of view may help explain the observed epidemiological trends in Khuzestan. Iran′s brucellosis control program in livestock relies on a combination of test‐and‐slaughter and vaccination policies. For small ruminants (sheep and goats), the Iranian Veterinary Organization uses the live attenuated Rev.1 vaccine, which has been shown to reduce the epidemic rate of brucellosis from 45% to 1.8% in vaccinated flocks [26, 27]. For cattle, the RB51 vaccine has been used since 2002, but its protective efficacy has been questioned; some studies have characterized RB51 as a vaccine strain with “very little protection.” The change of policy from S19 to RB51 is a major change of policy which could have altered the epidemiological trend of the disease. The marked fall in human cases reported in 2020–2021 (312 and 349 cases, respectively) needs to be considered carefully from a livestock perspective. There are several plausible explanations, not mutually exclusive. First, the COVID‐19 pandemic interrupted access to healthcare and surveillance activities and, as has been observed for other infectious diseases during this time, may have resulted in underdetection and underreporting of human brucellosis cases. Second, restrictions on movement during the pandemic may have reduced livestock trading and animal movements, temporarily interrupting transmission cycles. Thirdly, the economic impact of the pandemic might have affected livestock management (i.e., herd sizes or vaccination coverage).

The steep recovery since 2022 is associated with a series of changes in the livestock sector. The nationwide active surveillance program in industrial dairy cattle (2021–2024) demonstrated the presence of brucellosis despite vaccination [28]. Importantly, adult sheep and goats are not included in Iran′s vaccination component of the brucellosis control program—only lambs and kids aged 4–12 months are vaccinated—leaving a substantial portion of the adult small ruminant population susceptible [27]. Vaccination coverage in Iran has been described as inadequate to control brucellosis, particularly on traditional farms where the protective effects of vaccination are most needed [27]. In Khuzestan specifically, the Northern Highlands (Zagros foothills) represent 62.7% of human cases and are traditional livestock farming systems with possibly suboptimal vaccination coverage. The steep relative increase observed in the Southern Coastal zone may be due to livestock movements from high‐burden highland areas to coastal areas or to the expansion of informal livestock trading that bypasses veterinary controls.

Finally, a major limitation of this study is the lack of population denominators for age, sex, residence, and geographical zone. Therefore, subgroup analyses were performed on reported cases, rather than incidence rates. The temporal patterns seen here, therefore, reflect changes in disease burden and case distribution as reported and should not be interpreted as direct measures of changes in risk in the population. Future studies with population data that include subgroup information would allow estimation of incidence rates and a better evaluation of risk in demographic and geographic subgroups.

## 5. Conclusions

This study demonstrates a marked resurgence of human brucellosis in Khuzestan Province, southwestern Iran, beginning in 2021 and continuing through 2024, with an average annual increase of 10.7% in crude incidence. Reported case counts rose in all demographic categories, with older adults showing the largest increases, whereas rural communities and the Northern Highlands consistently saw the highest number of cases. Statistically significant temporal changes were observed; however, these findings are based upon surveillance data aggregated by population and should be interpreted as variations in reported case counts, not disease risk by subgroup. Results emphasize the need to strengthen One Health control measures, including increased livestock vaccination, strong surveillance and early diagnosis, and targeted public health education in high‐burden areas. Investment in integrated human and animal health programs is the key to reducing the burden of brucellosis in the province.

## Author Contributions

Mehran Varnasseri Ghandeali, Maryam Dadar, and Homayoun Amiri conceptualized and supervised the study. Maryam Dadar designed the methodology. Amirmansoor Varnasseri Ghandali and Nazafarin Varnasseri acquired and curated the data. Mehran Varnasseri Ghandeali, Maryam Dadar, and Homayoun Amiri performed the statistical analyses, interpreted the results, and drafted the original manuscript. All authors reviewed and revised the final version of the manuscript.

## Funding

No funding was received for this manuscript.

## Disclosure

All authors read and approved the final manuscript.

## Ethics Statement

The research proposal was reviewed and approved by the Ethical and Protocol Review Committee of the College of Ahvaz Jundishapur University of Medical Sciences (Code: IR.AJUMS.REC.1399.011). All experiments were conducted in accordance with good laboratory practice guidelines.

## Conflicts of Interest

The authors declare no conflicts of interest.

## Data Availability

Data are available upon request due to privacy/ethical restrictions.

## References

[1] Laine C. G. , Johnson V. E. , Scott H. M. , and Arenas-Gamboa A. M. , Global Estimate of Human Brucellosis Incidence, Emerging Infectious Diseases. (2023) 29, no. 9, 1789–1797, 10.3201/eid2909.230052, 37610167. 37610167 PMC10461652

[2] Grace D. and Cook E. , Sing A. , Zoonoses and Poverty: The Multiple Burdens of Zoonoses in Low- and Middle-Income Countries, Zoonoses: Infections Affecting Humans and Animals, 2023, Springer, 1685–1697, 10.1007/978-3-031-27164-9_46.

[3] Pakzad R. , Pakzad I. , Safiri S. , Shirzadi M. R. , Mohammadpour M. , Behroozi A. , Sullman M. J. , and Janati A. , Spatiotemporal Analysis of Brucellosis Incidence in Iran from 2011 to 2014 Using GIS, International Journal of Infectious Diseases. (2018) 67, 129–136, 10.1016/j.ijid.2017.10.017. 29122689

[4] Dadar M. , Shahali Y. , and Fakhri Y. , A Primary Investigation of the Relation Between the Incidence of Brucellosis and Climatic Factors in Iran, Microbial Pathogenesis. (2020) 139, 103858, 10.1016/j.micpath.2019.103858, 31712119. 31712119

[5] Iranian Statistical Center , Khūzestān (Province, Iran) - Population Statistics, Charts, Map and Location, 2026, Statistical Centre of Iran, http://www.citypopulation.de/en/iran/prov/admin/06__kh%25C5%25ABzest%25C4%2581n/.

[6] Mirnejad R. , Jazi F. M. , Mostafaei S. , and Sedighi M. , Epidemiology of Brucellosis in Iran: A Comprehensive Systematic Review and Meta-Analysis Study, Microbial Pathogenesis. (2017) 109, 239–247, 10.1016/j.micpath.2017.06.005, 28602839. 28602839

[7] Pordanjani S. R. , Moradi G. , Pourmohammadi B. , Mazaheri E. , Farivar F. , and Derakhshan S. , Spatiotemporal Epidemiology of Brucellosis in Iran From 2009 to 2018: A Mixed Ecological Study, Journal of Research in Medical Sciences. (2025) 30, no. 1, 10.4103/jrms.jrms_728_23, 40822563. PMC12352691 40822563

[8] Golshani M. and Buozari S. , A Review of Brucellosis in Iran: Epidemiology, Risk Factors, Diagnosis, Control, and Prevention, Iranian Biomedical Journal. (2017) 21, no. 6, 349–359, 28766326. 28766326 10.18869/acadpub.ibj.21.6.349 PMC5572431

[9] Norouzinezhad F. , Erfani H. , Norouzinejad A. , Ghaffari F. , and Kaveh F. , Epidemiological Characteristics and Trend in the Incidence of Human Brucellosis in Iran From 2009 to 2017, Journal of Research in Health Sciences. (2021) 21, no. 4, e00535, 10.34172/jrhs.2021.70, 36511231. 36511231 PMC8957668

[10] Asban P. , Kiani F. , Mohammadi M. J. , Ghanbari S. , Amiri H. , and Bichast R. K. B. , Investigated of Epidemiological and Incidence of Human Brucellosis in Southwest of Iran, a Retrospective Study From 2014 to 2021, Archives of Clinical Infectious Diseases. (2024) 19, no. 5, e150500, 10.5812/archcid-135931.

[11] Liu Z. , Gao L. , Wang M. , Yuan M. , and Li Z. , Long Ignored but Making a Comeback: A Worldwide Epidemiological Evolution of Human Brucellosis, Emerging Microbes & Infections. (2024) 13, no. 1, 2290839, 10.1080/22221751.2023.2290839, 38039063. 38039063 PMC10878345

[12] Lai S. , Chen Q. , and Li Z. , Human Brucellosis: An Ongoing Global Health Challenge, China CDC Weekly. (2021) 3, no. 6, 120–123, 10.46234/ccdcw2021.031, 34595017. 34595017 PMC8393116

[13] Liu Z. , Ye Y. , Zheng C. , Shi Y. , Xue C. , Yuan M. , Li Z. , and Diao L. , Spatiotemporal Epidemiological Characteristics of Human Brucellosis in Henan Province, From 1956 to 2023, BMC Public Health. (2025) 25, no. 1, 10.1186/s12889-025-23182-5, 41063091. PMC12506304 41063091

[14] Bagheri H. , Tapak L. , Karami M. , Amiri B. , and Cherghi Z. , Epidemiological Features of Human Brucellosis in Iran (2011-2018) and Prediction of Brucellosis With Data-Mining Models, Journal of Research in Health Sciences. (2019) 19, no. 4, e00462, 32291361. 32291361 PMC7183567

[15] Alizadeh-Barzian K. , Nikkhooy Z. , Soltani R. , Jamshidi M. , Sabaghan M. , Difrakhsh A. , Ghanavati R. , Parash M. , and Jamshidi A. , Epidemiologic, Demographic, and Clinical Characteristics of Brucellosis in Behbahan County, Southwest Iran, During the Years 2000-2021, Jorjani Biomedicine Journal. (2024) 12, no. 4, 22–25.

[16] Kashfi M. , Hatamian N. , and Rakhshani T. , Epidemiological Study of the Brucellosis in Iran, Andimeshk, 2001-2016, Journal of Health Sciences & Surveillance System. (2018) 6, no. 1, 23–28.

[17] Seraji M. S. , Charati J. Y. , Mahmoudi F. B. , Tahamtan R. A. M. , Vahedi H. , and Shojaei J. , Epidemiology of Brucellosis in Mazandaran, North of Iran in a Nine-Year Period (2009-2017), Caspian, Journal of Internal Medicine. (2024) 15, no. 4. 10.22088/cjim.15.4.666 PMC11444106 39359437

[18] Sadooghi N. , Alamian S. , Moghadam H. G. , Yazdanmanesh M. , and Dadar M. , Serological and Bacterial Prevalence of *Brucella* spp. in Suspected Patients: A Risk Factor Analysis in North Khorasan, Iran, Journal of Microbiology. (2024) 16, no. 5, 639–647, 10.18502/ijm.v16i5.16797, 39534295. PMC11551654 39534295

[19] Amanpour S. and Moradi H. , Spatial Analysis and Distribution of Brucellosis in Urban and Rural Areas (Case Study of Ilam Province), Sustainable Development of Geographical, Environment. (2023) 4, no. 7, 15–31.

[20] Kassiri H. , Amani H. , and Lotfi M. , Epidemiological, Laboratory, Diagnostic and Public Health Aspects of Human Brucellosis in Western Iran, Asian Pacific Journal of Tropical Biomedicine. (2013) 3, no. 8, 589–594, 10.1016/S2221-1691(13)60121-5. 23905014 PMC3703550

[21] Honarvar B. , Moghadami M. , Lankarani K. , Davarpanah M. , Ataolahi M. , Farbod A. , Eskandari E. , Panahi M. , Ghorbani A. , and Zahiri Z. , Brucellosis as a Neglected Disease in a Neglected Population: A Seroepidemiological Study of Migratory Nomads in the Fars Province of Iran, Epidemiology & Infection. (2017) 145, no. 3, 491–497, 10.1017/S0950268816002600, 27866494. 27866494 PMC9507681

[22] Mersini K. , Alla L. , Juma A. , Koleci X. , Crilly J. , and Bino S. , Review of Brucellosis in Albania: Disease Frequency in Humans and Animals, and One Health Efforts to Control the Disease, 1925 to Present, International Journal of Infectious Diseases. (2019) 79, no. 2019, 3–4, 10.1016/j.ijid.2018.11.028.

[23] Nayak G. , Panda C. , and Samal K. C. , Brucellosis: An Infectious Outbreak in China and New Threat to Civilization After COVID-19, Biotica Research Today. (2020) 2, no. 10, 1043–1045.

[24] Hamyali-Ainvand M. and Ebrahimi M. , Incidence Trend Analysis of Human Brucellosis in the Context of Human Development: An Ecological Study Using Joinpoint Regression in Iran, BMC Public Health. (2025) 25, no. 1, 10.1186/s12889-025-25304-5, 41250083. PMC12625387 41250083

[25] Dadar M. , Tiwari R. , Sharun K. , and Dhama K. , Importance of Brucellosis Control Programs of Livestock on the Improvement of One Health, Veterinary Quarterly. (2021) 41, no. 1, 137–151, 10.1080/01652176.2021.1894501. 33618618 PMC7946044

[26] Bahmani N. and Bahmani A. , A Review of Brucellosis in the Middle East and Control of Animal Brucellosis in an Iranian Experience, Reviews and Research in Medical Microbiology. (2022) 33, no. 1, e63–e69, 10.1097/mrm.0000000000000266.

[27] Dadar M. , Alamian S. , Bahreinipour A. , and Amiry K. , Epidemiology of Brucellosis in Sheep and Goats Across Different Production Systems in Regions With High Human Brucellosis Prevalence, Research in Veterinary Science. (2026) 208, 106235, 10.1016/j.rvsc.2026.106235, 42085969. 42085969

[28] Alamian S. , Amiry K. , Bahreinipour A. , and Dadar M. , Epidemiological Trends and Strain Distribution of Bovine Brucellosis in Vaccinated Industrial Dairy Cattle (2021–2024), Veterinary Medicine International. (2026) 2026, no. 1, 7432813, 10.1155/vmi/7432813, 42148180. 42148180 PMC13179448

